# Altered expression of exon 6 deleted progesterone receptor variant mRNA between normal human breast and breast tumour tissues

**DOI:** 10.1038/sj.bjc.6690366

**Published:** 1999-05-01

**Authors:** E Leygue, H Dotzlaw, P H Watson, L C Murphy

**Affiliations:** Departments of Biochemistry and Molecular Biology, andPathology, University of Manitoba, Faculty of Medicine, 770 Bannatyne Avenue, Winnipeg, Manitoba, Canada R3E 0W3

**Keywords:** progesterone receptor, breast cancer, tumour progression, variant mRNA

## Abstract

The progesterone receptor (PR) is an important prognostic marker in breast cancer as well as a marker of responsiveness to endocrine therapies. The presence of several exon-deleted PR variant mRNAs in both normal and neoplastic breast samples has recently been reported. Amongst them, a variant mRNA deleted in exon 6 (D6-PR mRNA) that if translated would encode a truncated PR-like protein missing the hormone binding domain and one of the transactivating domains of the wild-type PR protein. In order to determine whether changes in D6-PR variant expression could occur during tumorigenesis, its expression was investigated by reverse transcription and polymerase chain reaction in ten normal reduction mammoplasty samples, nine breast tumours with high PR levels (> 100 fmol mg^−1^ protein) and eight breast tumours with low PR levels (< 15 fmol mg^−1^ protein), as determined by ligand binding assay. The relative expression of D6-PR to wild-type PR mRNA was lower (*P* < 0.01) in normal than in all tumour breast samples. Moreover, a trend to lower (*P* < 0.1) relative D6-PR expression was observed in high PR tumours, compared to low PR tumours. These data suggest that increased expression of D6-PR occurs during tumorigenesis. © 1999 Cancer Research Campaign


*British Journal of Cancer* (1999) **80**(3/4), 379–382
© 1999 Cancer Research Campaign
Article no. bjoc.1998.0366

### Altered expression of exon 6 deleted progesterone receptor variant mRNA between normal human breast and breast tumour tissues

**E Leygue[1], H Dotzlaw[1], PH Watson[2] and LC Murphy[1]**

Departments of [1]Biochemistry and Molecular Biology and [2]Pathology, University of Manitoba, Faculty of Medicine, 770 Bannatyne Avenue, Winnipeg, Manitoba, Canada R3E 0W3

**Summary** The progesterone receptor (PR) is an important prognostic marker in breast cancer as well as a marker of responsiveness to endocrine therapies. The presence of several exon-deleted PR variant mRNAs in both normal and neoplastic breast samples has recently been reported. Amongst them, a variant mRNA deleted in exon 6 (D6-PR mRNA) that if translated would encode a truncated PR-like protein missing the hormone binding domain and one of the transactivating domains of the wild-type PR protein. In order to determine whether changes in D6-PR variant expression could occur during tumorigenesis, its expression was investigated by reverse transcription and polymerase chain reaction in ten normal reduction mammoplasty samples, nine breast tumours with high PR levels (> 100 fmol $mg^{-1}$ protein) and eight breast tumours with low PR levels (< 15 fmol $mg^{-1}$ protein), as determined by ligand binding assay. The relative expression of D6-PR to wild-type PR mRNA was lower ($P < 0.01$) in normal than in all tumour breast samples. Moreover, a trend to lower ($P < 0.1$) relative D6-PR expression was observed in high PR tumours, compared to low PR tumours. These data suggest that increased expression of D6-PR occurs during tumorigenesis.

**Keywords:** progesterone receptor; breast cancer; tumour progression; variant mRNA

The progesterone receptor (PR) is an important prognostic marker in breast cancer (Horwitz et al, 1975). Oestrogen receptor (ER)-positive breast tumours that also contain PR are considered 'good prognosis' tumours and are likely to respond to endocrine therapies (Horwitz et al, 1978). In contrast, absence of PR often characterizes 'poor prognosis' tumours (ER + PR–) and resistance to endocrine therapy (McGuire et al, 1991).

Similar to other members of the steroid receptor superfamily, PR is divided into structural domains (A–E) (Figure 1), the functions of which are widely documented (Tsai and O'malley, 1994). Two different PR isoforms, PR-A and PR-B, that are encoded by mRNAs transcribed from the same gene under the control of two different promoters (Kastner et al, 1990), have been identified in both normal and neoplastic tissues (Figure 1). The presence of several exon-deleted PR variant mRNAs in both normal and neoplastic breast samples has recently been reported (Leygue et al, 1996*a*; Richer et al, 1998; Yeates et al, 1998). Amongst them was a variant mRNA deleted in exon 6 (D6-PR mRNA) that, if translated, would encode a PR-A-like and/or a PR-B-like protein containing a truncated E domain. This variant, missing the hormone binding domain and one of the transactivating domains (AF-2) of the wild-type (WT) PR protein, has been shown to act in-vitro as a dominant-negative transcriptional inhibitor of PR-A and PR-B isoforms (Richer et al, 1998). Because expression of analogous exon-deleted or truncated variants has been associated with tumour progression in the case of ER variant mRNAs (for review see Murphy et al, 1997), it was of interest to determine whether D6-PR variant expression was also modified during tumorigenesis. In this study, we investigated D6-PR variant expression by reverse transcription polymerase chain reaction (RT-PCR) in ten normal reduction mammoplasties samples, nine breast tumours with high PR levels (considered 'good prognostic' tumours) and eight breast tumours with low PR levels (considered 'poor prognosis' tumours).

*Received 3 June 1998*
*Revised 28 October 1998*
*Accepted 4 November 1998*

*Correspondence to:* E Leygue

### MATERIALS AND METHODS

### Human breast tissues

Human breast specimens (27 cases) were from the Manitoba Breast Tumor Bank (Winnipeg, Manitoba, Canada). The processing of specimens collected in the Manitoba Breast Tumor Bank has already been described (Hiller et al, 1996). Briefly, each specimen was rapidly frozen as soon as possible after surgical removal. A portion of the frozen tissue block was processed to create a paraffin-embedded tissue block matched and orientated relative to the remaining frozen block. These paraffin blocks provide high quality histologic sections, which are used for pathologic interpretation and assessment and are mirror images of the frozen sections used for RNA extractions. For each case, tumour and normal tissues were histologically characterized by observation of paraffin sections. The presence of normal ducts and lobules as well as the absence of any proliferative lesion were confirmed in all ten normal reduction mammoplasties specimens. The 17 primary invasive ductal breast carcinomas were associated with high ER levels ranging from 105 to 386 fmol $mg^{-1}$ protein (mean = 190.7 fmol $mg^{-1}$ protein, standard deviation (s.d.) = 58.49), as determined by ligand binding assay. Within this group, nine tumours had a high PR level (PR > 100 fmol mg$^{-1}$ protein, mean = 156.4 fmol mg$^{-1}$ protein, s.d. = 28.4) and eight had a low PR level (PR < 15 fmol mg$^{-1}$ protein, mean = 8.6 fmol mg$^{-1}$ protein, s.d. = 4.6), as determined by ligand binding assay. The ages of patients associated with the tumour samples ranged from 37 to 91 (mean: 70 years old, s.d.: 14.4 years). For reduction mammoplasties, women were younger, with ages ranging from 19 to 41 years old (mean: 31.3 years old, s.d.: 8.3 years). Total RNA was extracted from frozen tissue and reverse transcribed in a final volume of 15 μl as previously described (Leygue et al, 1996*b*).

### PCR and identification of PCR products

The primers used consisted of D6U primer (5′-CTCT-CATTCAGTATTCTTGG-3′; sense; located in PR exon 5; 2989–23008) and D6L primer (5′-TGGGTTTGACTTCGTAGC-3′; antisense; located in PR exon 7; 3262–3245). The nucleotide positions correspond to published sequences of the human PR cDNA (Kastner et al, 1990). PCR amplifications were performed and PCR products analysed as previously described, with minor modifications (Leygue et al, 1996*b*). Briefly, 1 μl of reverse transcription mixture was amplified in a final volume of 10 μl, in the presence of 10 nmol$^{-1}$ [α-$^{32}$P] dCTP, 4 ng μl$^{-1}$ of each primer and 0.3 unit of *Taq* DNA polymerase. Each PCR consisted of 40 cycles (30 s at 60°C and 30 s at 94°C). As positive controls, aliquots of plasmid DNA containing previously (Leygue et al, 1996a) subcloned WT-PR (WT cont) or exon 6-deleted PR (D6 cont) sequences were amplified in parallel. PCR products were then separated on 6% polyacrylamide gels containing 7 M urea (PAGE). Following electrophoresis, the gels were dried and autoradiographed. The PCR product corresponding in size to D6-PR was subcloned and sequenced as previously described (Leygue et al, 1996*b*).

### Quantification and statistical analysis

The approach used to evaluate the exon-deleted variant mRNA expression relative to WT mRNA has been previously validated for exon-deleted ER variant mRNAs (Daffada et al, 1994; Leygue et al, 1996*b*). PCR co-amplification of WT and exon-deleted variant generates two bands whose ratio is constant with varying cycle number and is independent of initial input cDNA. This assay provides a semi-quantitative RT-PCR in which the internal control is the WT mRNA co-amplified and in which relative expression of variant mRNA can be determined for individual samples. Bands corresponding to D6-PR and WT mRNAs were excised from the gel and corresponding signals were subsequently measured after addition of 5 ml scintillant (ICN Pharmaceuticals, Inc., Irvine, CA, USA) by counting. The D6-PR signal was expressed as a percentage of the WT-PR signal. For each sample, three independent assays were performed and the mean determined. The statistical significance of differences in the relative levels of expression of D6-PR mRNAs was determined using the Mann–Whitney rank-sum test (two-sided).

### RESULTS

### Detection of D6-PR in all normal and tumour breast tissues

Total RNA from ten normal breast tissues and 17 breast tumour specimens was analysed by RT-PCR as described in Material and Methods using primers depicted in Figure 1. These primers were designed to allow the co-amplification of D6-PR and WT-PR mRNAs. Among the 17 tumours studied, nine had a high PR level (> 100 fmol mg$^{-1}$ protein) and eight had a low PR level (< 15 fmol mg$^{-1}$ protein), as

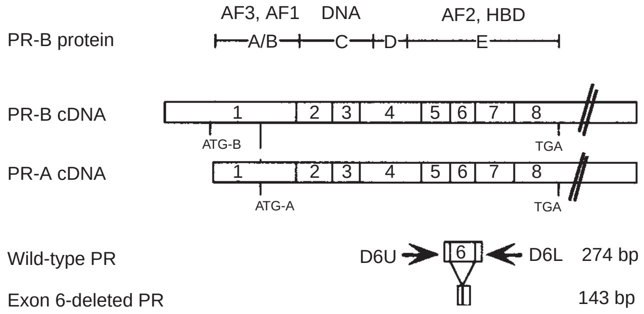

**Figure 1** Schematic representation of PR-B protein, PR-B and PR-A cDNAs and primers used to co-amplify WT-PR and D6-PR variant cDNAs. PR cDNAs contain eight different exons coding for a protein divided into structural and functional domains (A–E). A/B region of the receptor contains two transactivating domains (AF3 and AF1). The C region contains the DNA binding domain whereas region E, which is involved in hormone binding, contains another transactivating domain (AF2). ATG-B and ATG-A are the translational start sites of PR-B and PR-A proteins respectively. TGA, stop codon. D6U and D6L primers allow co-amplification of 274 bp and 143 bp fragments corresponding to WT-PR and D6-PR mRNAs, respectively

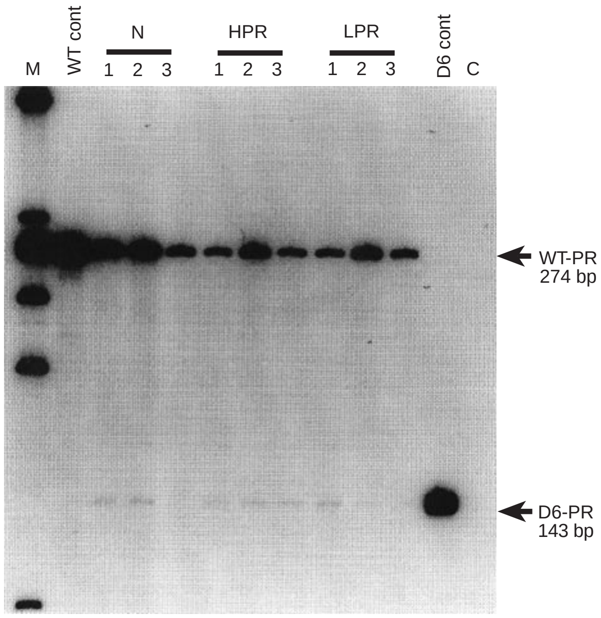

**Figure 2** Detection of exon 6-deleted PR variant mRNA in all human breast samples. Total RNA extracted from normal (N1–3), high PR tumour (HPR1–3) and low PR tumour (LPR1–3) breast tissue samples was reverse transcribed and PCR-amplified as described in Material and Methods, using D6U and D6L primers. Radioactive PCR products were separated on a 6% acrylamide gel and visualized by autoradiography. Bands that migrated at 274 bp and 143 bp were identified as corresponding to WT-PR and exon 6-deleted PR variant mRNAs, respectively. Plasmids containing either WT-PR (WT cont) or exon 6-deleted PR (D6 cont) sequences were used as positive control. M: molecular weight marker (φ× 174 HaeIII digest, Gibco BRL, Grand Island, NY, USA). C: no cDNA added during the PCR reaction

© Cancer Research Campaign 1999

determined by ligand binding assay. In each sample, two major bands that corresponded in size to that expected for WT-PR and D6-PR PCR products were obtained. Figure 2 presents a typical autoradiograph after one night's exposure. It should be noted that a longer exposure or addition of intensifying screens allowed the detection of D6-PR in both lanes N3 and LPR3 (data not shown). One should also note the presence, in samples where WT-PR signal is high (such as in lane N2, Figure 2), of some minor PCR products, that are not reproducibly obtained and therefore were not further characterized. The PCR product corresponding in size to D6-PR and reproducibly obtained was subcloned and subsequently sequenced. Sequence analysis showed the expected perfect junction between exon 5 and exon 7 (data not shown).

### Comparison of D6-PR variant expression in normal and tumour tissues

The D6-PR variant mRNA expression relative to WT-PR was then evaluated in each sample. It has been previously demonstrated that the co-amplification of WT and exon-deleted variant transcripts led to the synthesis of two PCR products. Further, the ratio of the signals obtained from these two products could be used to compare relative exon-deleted variant expression within samples (Daffada et al, 1994; Leygue et al, 1996*b*). The signal corresponding to D6-PR was expressed as a percentage of the WT-PR signal and the mean of three different assays calculated (Figure 3). The level of exon 6-deleted variant mRNA relative to the WT-PR mRNA was found to be significantly ($P < 0.05$) lower in normal (median = 4.8%) than in neoplastic breast tissues having either high PR or low PR (median = 9.19% and median = 25.13% respectively). The significance became higher ($P < 0.01$) when the tumour subgroups were considered together (median = 13.86%). Moreover, even though the difference did not achieve statistical significance ($0.1 < P < 0.05$), D6-PR relative expression appeared lower in tumours with high PR levels (median = 9.19%) than in tumours with low PR levels (median = 25.13%).

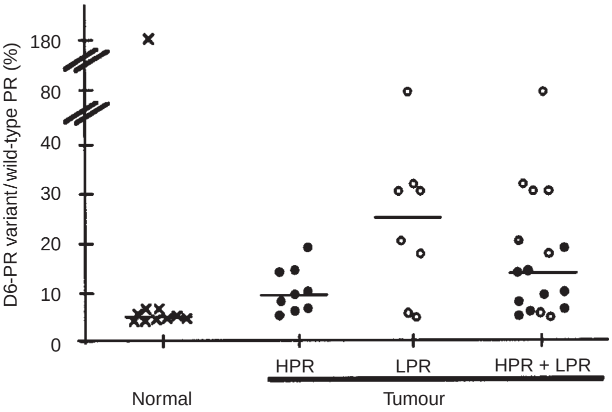

**Figure 3** Comparison of D6-PR relative expression between normal and tumour samples. Total RNA extracted from ten normal breast samples (×), nine tumours with high PR (●, HPR) and eight tumours with low PR (○, LPR) was reverse transcribed and PCR-amplified as described in Material and Methods, using D6U and D6L primers. D6-PR corresponding signal was measured as described in Material and Methods and expressed as a percentage of wild-type PR corresponding signal. For each sample, the mean of three different experiments is indicated. Bars: medians

### DISCUSSION

This study shows that the relative expression of D6-PR variant mRNA is altered between normal breast tissue and breast cancer tissue samples. The expression of D6-PR variant mRNA relative to WT-PR mRNA was investigated using a previously described semi-quantitative RT-PCR assay (Daffada et al, 1994; Leygue et al, 1996*b*). This assay allows the determination of the expression of PR variant mRNA relative to WT-PR mRNA using the very small amount of starting material provided by histopathologically well-characterized regions within human breast tissue.

It is important to establish whether or not this differential expression of D6-PR variant is maintained at the protein level. Anti-PR antibodies available to date (such as hPRa-7; Clarke et al, 1987), while able to recognize the predicted PR variant proteins, cannot provide any information on the unique primary sequence of the recognized molecule (Yeates et al, 1998). We are in the process of developing an antibody, raised against the predicted 12 specific C-terminal amino acids of the D6-PR protein (Leygue et al, 1996*a*) that could be used for the specific immunochemical detection of D6-PR variant protein within paraffin-embedded breast tissue sections or by Western blotting.

The recombinant D6-PR protein, has recently been shown to bind constitutively the progesterone receptor element (PRE) DNA consensus sequence and to exhibit dominant-negative activity on PR-A-and PR-B-induced transcription (Richer et al, 1998). Interestingly, a naturally occurring ER variant mRNA deleted in exon 7 and encoding an analogous truncated molecule lacking the hormone binding domain of the WT-ER can also act as a dominant-negative regulator of WT-ER, at least under some circumstances (Wang and Miksicek, 1991; Fuqua et al, 1992).

Relative levels of some of the ER variants were found to be increased during tumour progression. Exon 7-deleted variant mRNA level was shown to be higher in ER + PR – versus ER + PR + tumours (Fuqua et al, 1992). Exon 5-deleted ER variant mRNA expression was found higher in ER – PR + versus ER + PR + tumours (Fuqua et al, 1991) and was decreased in normal versus tumour breast tissues (Leygue et al, 1996*b*). It has thus been speculated that expression of these ER variants may be altered during breast tumorigenesis and progression and may have a role in progression from hormone dependence to independence in breast cancer (Murphy et al, 1997). This aspect of tumour progression consists of altered oestrogen signalling, the acquisition of resistance to the cytostatic effects of the anti-oestrogen tamoxifen and subsequently in the failure to respond to agents such progestins and probably antiprogestins (RU 486) (Horwitz et al, 1995). The apparent lower relative expression of D6-PR in normal breast samples compared to tumour tissues, as well as in high PR tumours compared to low PR tumours, is therefore of interest, since normal tissue, high PR tumours and low PR tumours may represent steps in tumour progression which correlate with increasing relative D6-PR expression. In order to clarify such issues, larger numbers of samples require screening for D6-PR expression.

The measurement of PR is an important tool in clinical decision-making with respect to prognosis and treatment of human breast cancer. Furthermore, the level of PR expression provides important clinical information as shown by Clark et al (1983). As the use of enzyme-linked immunosorbent assays (ELISA) and immunohistochemical assays for PR detection increases, it is likely that variant PR expression will interfere with these assays. Such

© Cancer Research Campaign 1999

capability of variant forms of receptor to possibly interfere with immunohistochemical detection of the WT molecule has recently been demonstrated for ER (Huang et al, 1997 and unpublished data).

In conclusion, we show in this study that exon 6-deleted PR variant mRNA relative expression is increased during breast tumorigenesis. We speculate that PR variants may have a role in tumorigenesis and/or be a marker of breast cancer progression, as already suggested for ER variants.

### ACKNOWLEDGEMENTS

This work was supported by grants from the Canadian Breast Cancer Research Initiative (CBCRI) and the US Army Medical Research and Materiel Command (USAMRMC). The Manitoba Breast Tumor Bank is supported by funding from the National Cancer Institute of Canada (NCIC). EL is a recipient of a USAMRMC Postdoctoral Fellowship, PHW is a Medical Research Council of Canada (MRC) Clinician-Scientist, LCM is an MRC Scientist.

### REFERENCES

Clark GM, McGuire WL, Hubay CA, Pearson OH and Marshall JS (1983) Progesterone receptors as a prognostic factor in stage II breast cancer. *N Engl J Med* **309**: 1343–1347

Clarke CL, ZainoRJ, Feil PD, Miller JV, Steck ME, Ohlsson-Wilhem BM and Satyaswaroop PG (1987) Monoclonal antibodies to human progesterone receptor: characterization by biochemical and immunohistochemical techniques. *Endocrinology* **121**: 1123–1132

Daffada AA, Johnston SRD, Nicholls J and Dowsett M (1994) Detection of wild type and exon 5-deleted splice variant oestrogen receptor (ER) mRNA in ER-positive and -negative breast cancer cell lines by reverse transcription/polymerase chain reaction. *J Mol Endocrinol* **13**: 265–273

Fuqua SA, Fitzgerald SD, Chamness Gc, Tandon AK, McDonnell DP, Nawaz Z, O'Malley BW and McGuire WL (1991) Variant human breast tumor estrogen receptor with constitutive transcriptional activity. *Cancer Res* **51**: 105–109

Fuqua SA, Fitzgerald SD, Allred DC, Elledge RM, Nawaz Z, McDonnell DP, O'Malley BW, Greene GL and McGuire WL (1992) Inhibition of estrogen receptor action by a naturally occurring variant in human breast tumors. *Cancer Res* **52**: 483–486

Hiller T, Snell L and Watson PH (1996) Microdissection/RT-PCR analysis of gene expression. *Biotechniques* **21**: 38–44

Horwitz KB, McGuire WL, Pearson OH and Segaloff A (1975) Predicting response to endocrine therapy in human breast cancer: a hypothesis. *Science* **189**: 726–727

Horwitz KB, Koseki Y and McGuire WL (1978) Estrogen control of progesterone receptor in human breast cancer: role of estradiol and antiestrogen. *Endocrinology* **103**: 1742–1751

Horwitz KB, Tung L and Takimoto GS (1995) Novel mechanisms of antiprogestin action. *J Steroid Biochem Mol Biol* **53**: 9–17

Huang A, Leygue E, Snell L, Murphy LC and Watson PH (1997) Expression of estrogen receptor variant messenger RNAs and determination of estrogen receptor status in human breast cancer. *Am J Pathol* **150**: 1827–1833

Kastner P, Krust A, Turcotte B, Stropp U, Tora L, Gronemeyer H and Chambon P (1990) Two distinct estrogen-regulated promoters generate transcripts encoding the two functionally different human progesterone receptor forms A and B. *Embo J* **9**: 1603–1614

Leygue E, Dotzlaw H, Watson PH and Murphy LC (1996*a*) Identification of novel exon-deleted progesterone receptor variant mRNAs in human breast tissue. *Biochem Biophys Res Commun* **228**: 63–68

Leygue E, Watson PH and Murphy LC (1996*b*) Estrogen receptor variants in normal human mammary tissue. *J Natl Cancer Inst* **88**: 284–290

McGuire WL, Chamness GC and Fuqua SA (1991) Estrogen receptor variants in clinical breast cancer. *Mol Endocrinol* **5**: 1571–1577

Murphy LC, Leygue E, Dotzlaw H, Douglas D, Coutts A and Watson PH (1997) Oestrogen receptor variants and mutations in human breast cancer. *Ann Med* **29**: 221–234

Richer JK, Lange CA, Wierman AM, Brooks KM, Tung L, Takimoto GS and Horwitz KB (1998) Progesterone receptor variants found in breast cells repress transcription by wild-type receptors. *Breast Cancer Res Treat* **48**: 231–241

Tsai MJ and O'Malley BW (1994) Molecular mechanisms of action of steroid/thyroid receptor superfamily members. *Annu Rev Biochem* **63**: 451–486

Wang Y and Miksicek RJ (1991) Identification of a dominant negative form of the human estrogen receptor. *Mol Endocrinol* **5**: 1707–1715

Yeates C, Hunt SM, Balleine RL and Clarke CL (1998) Characterization of a truncated progesterone receptor protein in breast tumors. *J Clin Endocrinol Metab* **83**: 460–467

 *© Cancer Research Campaign 1999*
